# RPS15A promotes gastric cancer progression via activation of the Akt/IKK‐β/NF‐κB signalling pathway

**DOI:** 10.1111/jcmm.14141

**Published:** 2019-01-19

**Authors:** Chenchen Liu, Xigan He, Xiaowen Liu, Jian Yu, Meng Zhang, Fudong Yu, Yanong Wang

**Affiliations:** ^1^ Department of Gastric Surgery Fudan University Shanghai Cancer Center Shanghai China; ^2^ Department of Oncology Shanghai Medical College, Fudan University Shanghai China; ^3^ Department of Hepatic Surgery Fudan University Shanghai Cancer Center Shanghai China; ^4^ Department of Oncology Rizhao Central Hospital Rizhao Shandong China; ^5^ Department of Pathology Fudan University Shanghai Cancer Center Shanghai China; ^6^ Key Laboratory of Reproduction Regulation of NPFPC, SIPPR, IRD Fudan University Shanghai China

**Keywords:** Epithelial‐mesenchymal transition, Gastric cancer, Metastasis, NF‐κB, Ribosomal protein S15A

## Abstract

This study aimed to investigate the clinical significance, potential biological function and underlying mechanism of RPS15A in gastric cancer (GC) progression. RPS15A expression was detected in 40 pairs of GC tissues and matched normal gastric mucosae (MNGM) using qRT‐PCR analysis. Immunohistochemistry assay was conducted using a tissue microarray including 186 primary GC samples to characterize the clinical significance of RPS15A. A series of in vitro and in vivo assays were performed to elucidate the biological function of RPS15A in GC development and underlying molecular mechanisms. The expression of RPS15A was significantly up‐regulated in GC samples compared to MNGM, and its expression was closely related to TNM stage, tumour size, differentiation, lymph node metastasis and poor patient survival. Ectopic expression of RPS15A markedly enhanced the proliferation and metastasis of GC cells both in vitro and in vivo. RPS15A overexpression also promoted the epithelial‐mesenchymal transition (EMT) phenotype formation of GC cells. Investigations of underlying mechanisms found that RPS15A activated the NF‐κB signalling pathway by inducing the nuclear translocation and phosphorylation of the p65 NF‐κB subunit, transactivation of NF‐κB reporter and up‐regulating target genes of this pathway. In addition, RPS15A overexpression activated, while RPS15A knockdown inhibited the Akt/IKK‐β signalling axis in GC cells. And both Akt inhibitor LY294002 and IKK inhibitor Bay117082 neutralized the p65 and p‐p65 nuclear translocation induced by RPS15A overexpression. Collectively, our findings suggest that RPS15A activates the NF‐κB pathway through Akt/IKK‐β signalling axis, and consequently promotes EMT and GC metastasis. This newly identified RPS15A/Akt/IKK‐β/NF‐κB signalling pathway may be a potential therapeutic target to prevent GC progression.

## 1 INTRODUCTION

Gastric cancer (GC) is one of the most aggressive malignancies with a high incidence and metastasis rate, accounting for an estimated annual 720 000 deaths worldwide.^1^ Despite important advances in diagnosis and therapeutic strategies, the 5‐year survival rate of GC, especially patients with metastatic GC, is still low.^2^ Therefore, identification and better understanding of novel biomolecules and signalling pathways involved in GC progression remain of great importance.

Like nearly all cancer types, metastasis represents the main cause of death in GC patients. The initial stage of metastatic progression is essentially dependent on the prominent biological event referred to as epithelial‐mesenchymal transition (EMT).^3^ During the EMT process, epithelial cells lose their junctions and apical‐basal polarity, reorganize their cytoskeletons, undergo a change in the signalling programs that define cell shape and reprogram gene expression.^4^ Biologically, EMT is a complex process that is typically driven by aberrant activation of transcription factors such as Slug, Snail, ZEB1, ZEB2 and Twist, as well as various signalling pathways, including NF‐κB, Wnt, Notch, Hedgehog, TGF‐β and others.^5^

The NF‐κB signalling pathway has been widely demonstrated as one of the most commonly activated and essential pathways for EMT and GC metastasis.^5^, ^6^, ^7^ The core of this pathway, transcription factor NF‐κB, consisting of two distinct subunits, p50 and p65, is subject to multiple levels of control. In non‐stimulated cells, NF‐κB is normally sequestered in the cytoplasm through interaction with the general inhibitors of NF‐κB (IκBs), the best characterized of which is IκB‐α. Upon activation of the pathway, IκB‐α is phosphorylated by IκB kinases (IKK).^8^ Such phosphorylation triggers IκB‐α degradation by ubiquitin‐mediated proteolysis, and promotes the release and relocalization of NF‐κB (p65/p50) to the nucleus, where it exerts its role in gene expression.^9^ Despite these multiple levels of tight regulation, however, molecular mechanisms causing constitutive activation of NF‐κB signalling are still largely unknown.

Ribosomal protein S15A (RPS15A), a member of the RPS family, maps to human chromosome 16p12.3 locus and encodes a highly conserved 40S ribosomal protein. RPS15A promotes the binding of capped mRNA to the small ribosomal subunit at the early stages of translation.^10^ RPS15A has also been identified as Ca2+/CaM binding partner modulating ribosome assembly and translation.^11^ Meanwhile, increasing evidence indicates that RPS15A, like other RPS family proteins, exhibits various extra‐ribosomal functions, such as cell division, tumourigenesis and progression.^12^ In response to induction of transforming growth factor‐β, RPS15A enhances cell growth of lung cancer.^13^, ^14^ Down‐regulation of RPS15A induces apoptosis and inhibits proliferation of human glioblastoma cells in vivo and in vitro via AKT pathway.^15^, ^16^ In addition, RPS15A promotes malignant transformation of colorectal cancer through misregulation of p53 signalling pathway.^17^ However, the clinical significance, potential biological function of RPS15A in GC progression and underlying mechanisms remain unclear.

In this study, we investigated the role of RPS15A in GC development. Our data revealed that RPS15A is significantly up‐regulated in GC tissues and associated with poor prognosis. By gain‐ and loss‐of‐function studies, we demonstrated that RPS15A promotes the proliferation, migration and invasion of GC cells both in vitro and in vivo. Mechanically, RPS15A activates the Akt/IKK‐β/NF‐κB signalling pathway to enhance EMT and GC progression.

## 2 MATERIALS AND METHODS

### 2.1 Patients and tissue samples

This study was approved by The Clinical Research Ethics Committee of Fudan University Shanghai Cancer Center (FUSCC, Shanghai, China). Written informed consent was obtained from all participants. A total of 40 GC tissues and matched normal gastric mucosae (MNGM) were obtained from the biobank of FUSCC. None of patients had received local or systemic treatment prior to the surgery. Tumour stage was classified according to the guidelines of the National Comprehensive Cancer Network 2010 (NCCN 2010). In addition, tissue microarrays (TMAs) including 186 primary GC samples were constructed as previously described.^18^

### 2.2 Cell lines

Human GC cell lines (AGS, MKN‐28, BGC‐823, HGC‐27, SGC‐7901, MGC‐803 and MKN‐45) and normal gastric epithelial cell line GES‐1 were obtained from the Shanghai Cell Bank, Chinese Academy of Sciences (Shanghai, China) and maintained in the recommended growth medium. All medium contained 10% foetal bovine serum (FBS) and 1% penicillin‐streptomycin. All cell lines were cultured in a humidified atmosphere of 5% CO_2_ at 37°C.

### 2.3 Plasmid construction and transfection

For RPS15A knockdown, the target sequence was sh‐1: 5ʹ‐AAA GAG AGG CAA ACG CCA GGT GC‐3ʹ; sh‐2: 5ʹ‐CCA AAG TCA TCG TCC GGT TTC TC‐3ʹ. pLenti‐hU6‐MCS‐CMV (Biolink, Shanghai, China) was used for shRNA plasmid construction. For RPS15A overexpression, RPS15A sequence was PCR‐amplified and subcloned into the pLOV‐EF1a‐EGFP‐P2A plasmid (Oobio, Shanghai, China) for lentivirus production. Plasmid sequences were confirmed by DNA sequencing before use. The transfection was performed using Lipofectamine 2000 reagent (Invitrogen, Carlsbad, CA, USA).

### 2.4 Quantitative real‐time PCR (qRT‐PCR), Western blotting, immunofluorescence assays

Quantitative real‐time PCR (qRT‐PCR), Western blotting and immunofluorescence assays were performed as previously described.^4^ The primers were described in Table S1.

### 2.5 Immunohistochemistry (IHC) analysis

Immunohistochemistry was performed on tissue microarrays including 186 primary GC samples. The staining score was calculated based on the intensity and extent of staining. Briefly, the staining intensity scores (0, negative; 1, weak; 2, moderate; 3, strong) and staining area scores (0, 0%‐5%; 1, 6%‐25%; 2, 26%‐50%; 3, >50%) were summed up to give a total score, and a total score ≥3 was considered as significant overexpression and noted as “RPS15A high” to simplify data analysis.

### 2.6 Cell proliferation assay

Indicated cells were seeded into 96‐well plates at a density of 2 × 10^3^/well for culture, and cell proliferation was measured using CCK‐8 reagents (Dojindo, Kumamoto, Japan). The staining intensity in the medium was documented every 24 hours for 5 days. Each experiment was repeated three times and five wells were used for each time‐point per group.

### 2.7 Colony formation assay

Cells were seeded into six‐well plates at a density of 1×10^3^ cells/well and cultured in complete medium at 37°C in 5% CO_2_ for 14 days. Growth medium was refreshed every three days. At the end of the experiment, the cells were fixed with 4% paraformaldehyde for 15 minutes, stained with Giemsa solution for 10 minutes and then photographed and calculated. A group of 50 cells or more was counted as a colony.

### 2.8 Wound‐healing assay

Cells were seeded in six‐well plates with complete medium and cultured until they reached confluence. Then a linear wound about 300‐500 μm wide was generated with a standard 200 μL pipette tip. Wounded monolayers were washed twice with 1xPBS to remove non‐adherent cells. Wound closure was examined and photographed at pre‐determined time‐points (0, and 48 hours) in five random microscopic regions.

### 2.9 Transwell invasion assay

About 5×10^4^ cells with FBS‐free medium were seeded into the upper chamber of 8.0 μm pore transwells (Millipore Corporation, USA) pre‐coated with Matrigel (BD Bioscience, USA). The medium supplemented with 10% FBS was added into the lower compartment as chemokine. After 36 hours incubation, the cells attached to the lower surface of the chamber were counted in five random fields after crystal violet staining.

### 2.10 In vivo tumour growth assay

The transduced cells (2×10^6^) were injected subcutaneously into the groin of 4‐ to 6‐week‐old male nude mice (n = 5 for each group) (Institute of Zoology, Chinese Academy of Sciences, Shanghai, China). Tumours were measured every 4 days, and tumour volumes were calculated using the following formula: Volume (mm^3^) = 4π/3 × (width/2)^2^ × (length/2). One month after inoculation, all mice were killed. Tumour xenografts were collected, photographed and weighed.

### 2.11 Lung metastasis model

A lung metastasis model was generated to validate the effect of RPS15A on GC metastatic ability in vivo by injecting the transduced cells (5 × 10^6^) into the tail veins of nude mice (n = 5 for each group). All mice were killed 6 weeks after operation, and the lungs were then removed for pathologic examination, H&E staining and count of the lung metastatic nodules. The animal study was performed according to the Animal Care Guidelines of FUSCC, Shanghai, China.

### 2.12 RNA‐seq and computational analysis

RNA‐seq was performed using Hiseq3000 (Illumina, USA). LifeScope v2.5.1 was used to align the reads to the genome, generate raw counts corresponding to each known gene and calculate the RPKM (reads per kilobase per million) values. KEGG enrichment was used for the pathway analysis.

### 2.13 Luciferase reporter assay

The response element of NF‐κB was subcloned into the pGM‐CMV‐Luc vector (Yeasen, Shanghai, China). The final constructs were confirmed by DNA sequencing. Firefly luciferase activity was normalized to that of Renilla luciferase. Luciferase activity was detected using the dual‐luciferase reporter assay system (Promega) according to the manufacturer's instructions.

### 2.14 Statistical analyses

Data were analysed using one‐way analysis of variance or Student's *t* test for comparison between groups. The protein expression levels and clinicopathological parameters were compared by *χ* ^2^ test. Survival curves were plotted with the Kaplan‐Meier method and compared using the log‐rank test. *P* < 0.05 with a two‐sided test was considered to be statistically significant. Statistical analyses were performed with GraphPad Prism software Version 5.0 (San Diego, CA, USA).

## 3 RESULTS

### 3.1 RPS15A is up‐regulated in GC patients and associated with poor prognosis

**Figure 1:**
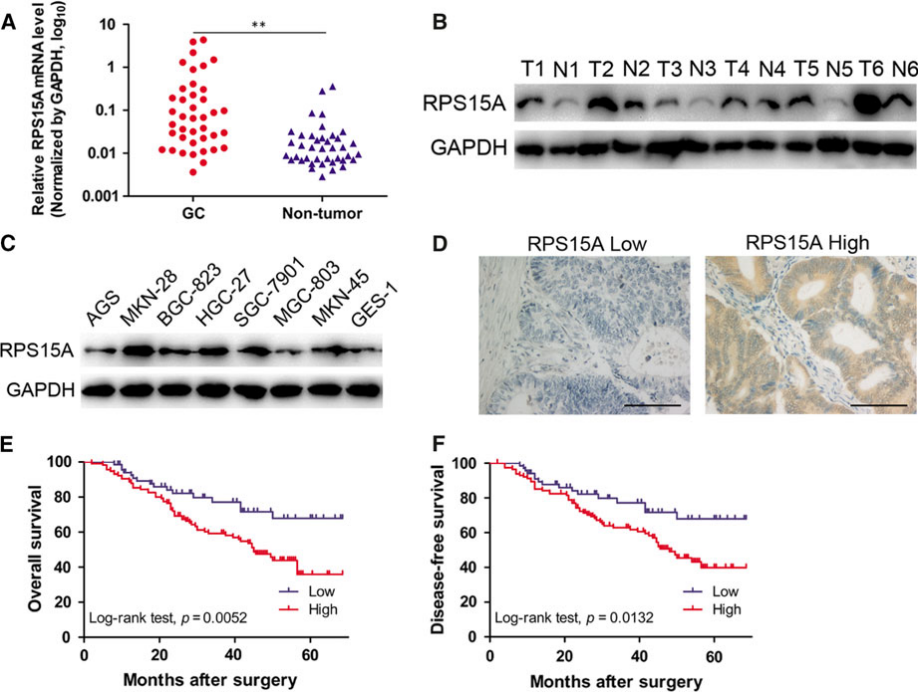
RPS15A is up‐regulated in GC patients and associated with poor prognosis. A, The mRNA levels of RPS15A in 40 GC tissues and paired normal gastric mucosae (PNGM) were determined by qRT‐PCR. GAPDH was used as an internal control. **P < 0.01 by Student's *t* test. B, The protein levels of RPS15A in six pairs of GC tissues (T1‐T6) and PNGM (N1‐N6) were evaluated by Western blotting. GAPDH was used as an internal control. C, RPS15A expression levels in normal gastric epithelial cell line GES‐1 and 7 GC cell lines were examined by Western blotting analysis. GAPDH was used as an internal control. D, Immunohistochemistry (IHC) assay of RPS15A expression was performed using a tissue microarray including 186 primary GC tissues. Representative IHC images showed high and low expression of RPS15A in GC tissues, respectively; scale bar = 200 μm. (E‐F) Influence of RPS15A expression patterns on overall survival (OS) and disease‐free survival (DFS) by Kaplan‐Meier analyses (Log‐rank test). Low expression of RPS15A, n = 70; high expression of RPS15A, n = 116

We first examined RPS15A mRNA levels in 40 pairs of GC and matched normal gastric mucosae (MNGM). The results showed that RPS15A mRNA was significantly increased in GC tissues compared with MNGM (Figure 1A). Similarly, up‐regulation of RPS15A in GC tissues was also confirmed at the translational level using Western blotting analysis (Figure 1B). In addition, we evaluated the RPS15A expression levels in normal gastric epithelial cell GES‐1 and a panel of GC cell lines. According to Western blotting results, elevated expression of RPS15A was observed in all seven GC cell lines compared to GES‐1 (Figure 1C). Taken together, these findings indicated that RPS15A is significantly up‐regulated in GC patients.

In order to determine the clinical significance of RPS15A overexpression in GC, immunohistochemistry analysis was performed using tissue microarrays including 186 primary GC samples. The immunostaining score of RPS15A was evaluated on the basis of staining intensity and extent, and all patients were categorized into high or low RPS15A expression group to simplify data analysis (Figure 1D). We then analysed the relationship between RPS15A expression and clinicopathological characteristics in GC patients, and found that RPS15A expression was closely correlated with aggressive phenotypes of GC, including TNM stage, tumour size, differentiation and lymph node metastasis (Table 1). Furthermore, Kaplan‐Meier analysis indicated that GC patients with higher RPS15A expression had markedly reduced overall survival (OS) and disease‐free survival (DFS) (Figure 1E,F). Subsequent multivariate Cox regression analysis demonstrated that elevated RPS15A expression remained an independent prognostic factor for poor OS and DFS of GC patients (Table 2). Collectively, RPS15A expression is significantly up‐regulated in GC patients and contributes to poor prognosis.

**Table 1:** Correlation between RPS15A expression and clinicopathological characteristics in 186 cases of gastric cancer tissues

Parameters	RPS15A expression		*P* value
	Low (n = 70)	High (n = 116)	
Age (y)			
<60	21	37	0.787
≥60	49	79	
Gender			
Male	46	64	0.157
Female	24	52	
Tumour size (cm)			
<2	50	66	**0.047**
≥2	20	50	
AJCC stage			
I‐II	51	46	**<0.001**
III‐IV	19	70	
Differentiation			
Well and moderate	49	19	**<0.001**
Poor	21	97	
Lymph node metastasis			
With (N1 + N2 + N3)	9	31	**0.026**
Without (N0)	61	85	

The bold number represents the *P*‐values with significant differences.

**Table 2:** Multivariate analysis of factors associated with overall survival (OS) or disease‐free survival (DFS) in gastric cancer

Variable	Multivariate analysis (OS)		Multivariate analysis (DFS)	
	HR (95% CI)	*P‐*value	HR (95% CI)	*P‐*value
Age (<60 vs ≥60 y)	1.005 (0.985‐1.026)	0.613	1.010 (0.990‐1.030)	0.322
Gender (male vs female)	1.212 (0.750‐1.959)	0.433	1.091 (0.667‐1.786)	0.729
Tumour size (≥2 vs <2 cm)	1.106 (0.763‐1.603)	0.594	1.205 (0.843‐1.723)	0.306
Differentiation (poor vs well and moderate)	1.097 (0.672‐1.793)	0.711	1.052 (0.640‐1.729)	0.841
AJCC stage (III‐IV vs I + II)	1.459 (0.750‐2.837)	0.266	1.594 (1.099‐2.313)	**0.014**
Lymph node metastasis (yes vs no)	1.721 (1.073‐2.759)	**0.024**	1.237 (0.844‐1.814)	0.275
RPS15A (high vs low)	1.763 (1.097‐2.833)	**0.019**	1.801 (1.135‐2.860)	**0.013**

Analyses were conducted using multivariate Cox proportional hazards regression. The bold number represents the *P*‐values with significant differences.

### 3.2 RPS15A enhances the malignant phenotypes of GC cells in vitro

**Figure 2:**
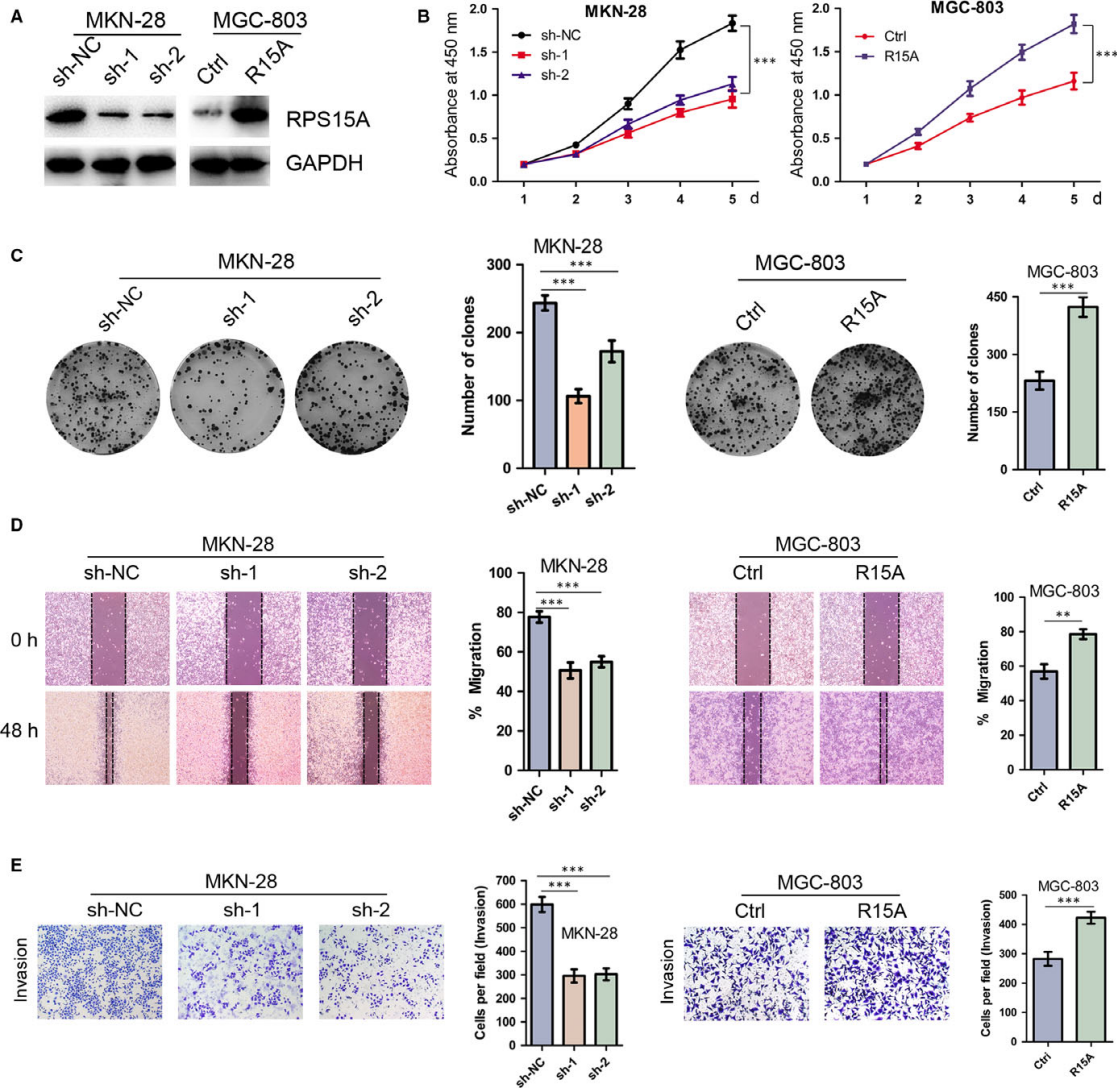
RPS15A promotes GC cell proliferation, migration and invasion in vitro. A, The RPS15A knockdown and overexpression effects were confirmed by Western blotting. GAPDH was used as an internal control. B, Depletion of RPS15A inhibited GC cell proliferation, whereas ectopic expression of RPS15A stimulated GC cell proliferation as determined by CCK‐8 assay. ***P < 0.001 by Student's *t* test. C, The effect of RPS15A on GC cell line colony formation. ***P < 0.001 by Student's *t* test. D, Indicated cells were subjected to scratch wound‐healing assay. The wound space was photographed at 0, and 48 h. The wound healing was measured with the following formula: 48‐h migration % = (0‐h width – 48‐h width of wound)/(0‐h width of wound). **P < 0.01; ***P < 0.001 by Student's *t* test. E, The transwell invasion assays showed that depletion of RPS15A obviously inhibited the invasion of MKN‐28 cells. Conversely, the ectopic expression of RPS15A promoted the invasion of MGC‐803 cells. The data are presented as the mean ± SD from three independent experiments. ***P < 0.001 by Student's *t* test

To assess the role of RPS15A in GC development, we performed loss‐ and gain‐of‐function studies in GC cells. We constructed a lentivirus vector harbouring shRNA‐RPS15A and established two stable knockdown MKN‐28 cell line which shows high RPS15A expression. Meanwhile, we stably overexpressed RPS15A in MGC‐803 cell line for its low RPS15A level (Figure 2A). CCK‐8 and colony formation assays demonstrated that the proliferation of GC cells was significantly inhibited after RPS15A silencing, while markedly increased by RPS15A overexpression (Figure 2B,C). Furthermore, wound‐healing assay showed that the migratory ability of GC cells was significantly impaired by RPS15A knockdown, while promoted by RPS15A overexpression (Figure 2D). Likewise, RPS15A knockdown significantly decreased the number of cells that invaded through Matrigel compared with the control cells. In contrast, RPS15A overexpression in MGC‐803 cells led to a significant increase in their invasive ability (Figure 2E). Taken together, RPS15A promotes the malignant phenotypes of GC cells in vitro.

### 3.3 RPS15A promotes GC cell growth and metastasis in vivo

**Figure 3:**
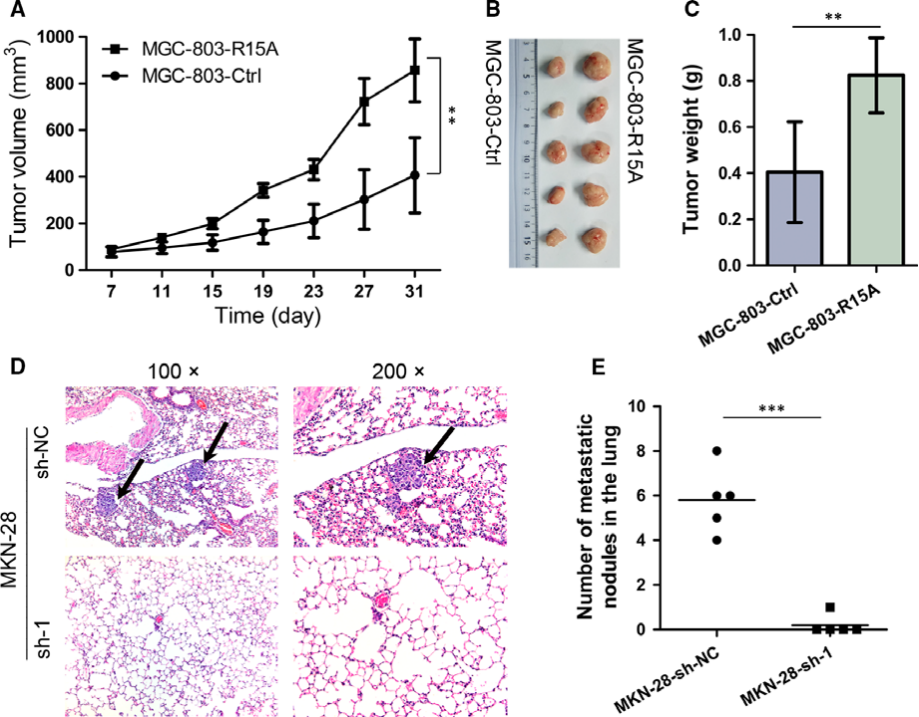
RPS15A promotes GC cell growth and metastasis in vivo. A, MGC‐803‐Ctrl and MGC‐803‐RPS15A cells (2×10^6^) were injected subcutaneously into the groin of nude mice (n = 5 for either group). Tumour volumes were measured on the indicated days. Error bars represent the mean ± SD. **P < 0.01 by Student's *t* test. (B‐C) One month after tumour cell injection, mice were killed. The representative xenograft tumours were shown (B) and the average tumour weight was measured (C). Error bars represent the mean ± SD. **P < 0.01 by Student's *t* test. D, MKN‐28‐sh‐NC and MKN‐28‐sh‐1 cells (5 × 10^6^) were injected into the tail vein of nude mice (n = 5 for either group) to establish a lung metastasis model. Representative results of H&E staining of lung metastatic nodules were shown. The metastatic nodules were indicated by arrows. E, The number of lung metastatic nodules was shown. ***P < 0.001 by Student's *t* test

To determine the effects of RPS15A on GC cell growth in vivo, xenograft tumour assay was performed in nude mice. As shown in Figure 3A, the tumours from RPS15A‐overexpressed MGC‐803 cells showed more active proliferative ability than the control group (n = 5 for each group). At the end‐point, the average tumour volume and weight were dramatically increased in MGC‐803‐RPS15A group compared to the control group (Figure 3A‐C). Overall, RPS15A promotes the tumourigenesis of GC in vivo.

To further investigate the in vivo effect of RPS15A on metastasis, we established a lung metastasis model by injecting stable MKN‐28‐knockdown and control cells into the tail veins of nude mice (n = 5 for each group). All mice were sacrificed 6 weeks after injection, and the lungs were then dissected out for histological analysis. As shown in Figure 3D,E, lung metastasis was found in 20.0% (1/5) of mice in the RPS15A‐silencing group compared with 100% (5/5) in the control group. In addition, a marked less number of lung metastatic nodules was observed in the RPS15A‐silencing group compared with the control group. These results indicate functional significance of RPS15A in GC metastasis.

### 3.4 RPS15A induces EMT in GC cells

**Figure 4:**
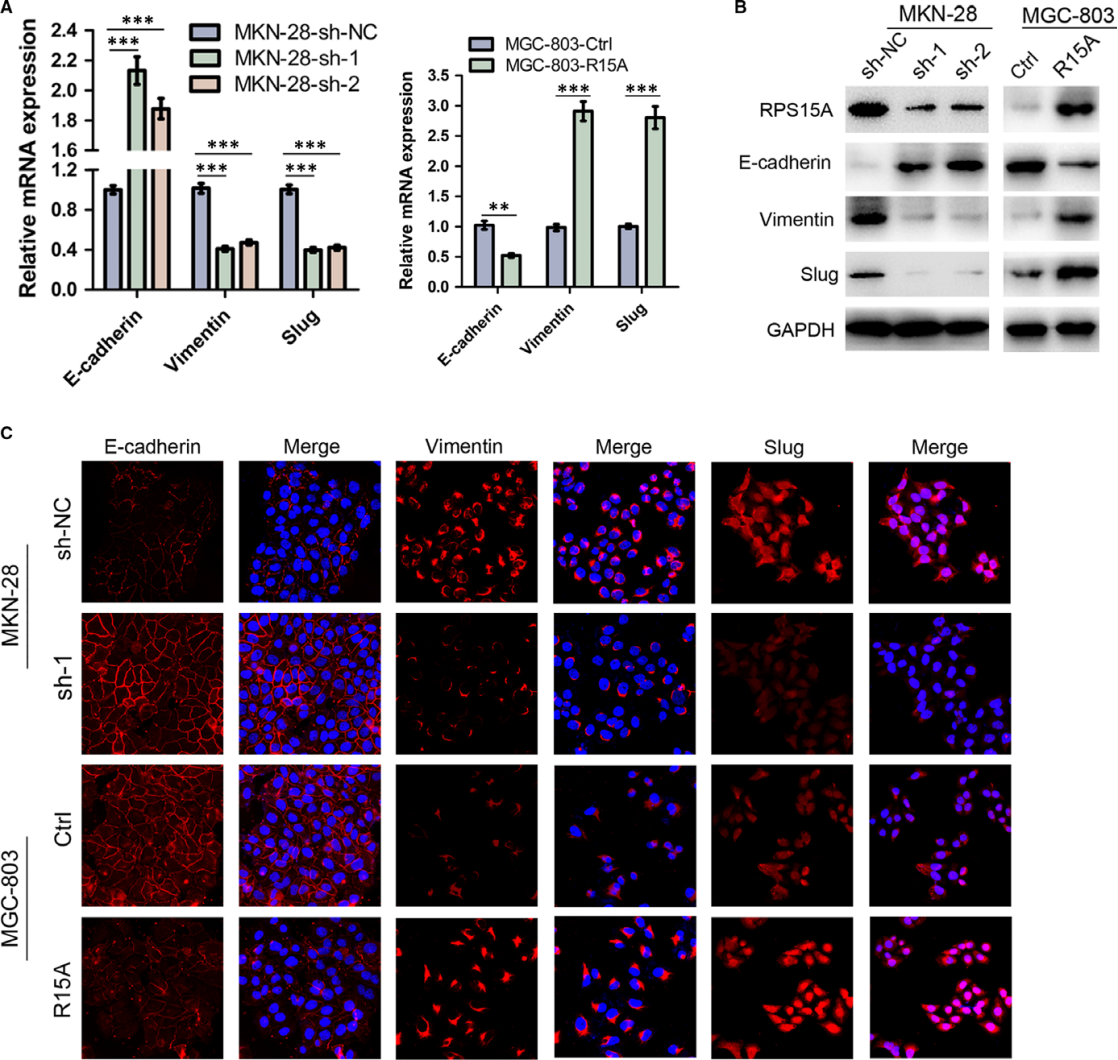
RPS15A regulated the expression of epithelial‐mesenchymal transition (EMT) markers in the GC cells. A, qRT‐PCR analysis of the expression of EMT markers (E‐cadherin, vimentin and slug) in indicated GC cells with modified RPS15A expression. GAPDH was used as an internal control. **P < 0.01; ***P < 0.001 by Student's *t* test. B, Western blotting analysis of the expression of EMT markers (E‐cadherin, vimentin and slug) in indicated GC cells with modified RPS15A expression. GAPDH was used as an internal control. C, Immunofluorescent staining of EMT markers (E‐cadherin, vimentin and slug) in indicated GC cells with modified RPS15A expression. Targeted proteins were stained in red colour, and the nuclei were stained with 4′,6‐diamidino‐2‐phenylindole (DAPI) in blue colour.

Given that epithelial‐mesenchymal transition (EMT) is considered a striking feature of most cancers and plays a crucial role in cancer metastasis and invasion,^5^ we then examined whether EMT might be an underlying mechanism for RPS15A‐induced GC metastasis. As shown in Figure 4A,B, in RPS15A‐overexpressed MGC‐803 cells, qRT‐PCR and Western blotting analyses revealed down‐regulation of the cohesive epithelial marker E‐cadherin and a corresponding up‐regulation of the mesenchymal markers vimentin and slug. Conversely, in RPS15A‐depleted MKN‐28 cells, E‐cadherin expression was elevated compared with the control cells, whereas vimentin and slug were down‐regulated (Figure 4A,B). In addition, the involvement of EMT was further supported by immunofluorescence assay (Figure 4C). Taken together, these findings demonstrated that RPS15A induces EMT in GC cells.

### 3.5 RPS15A promotes EMT through NF‐κB signalling

**Figure 5:**
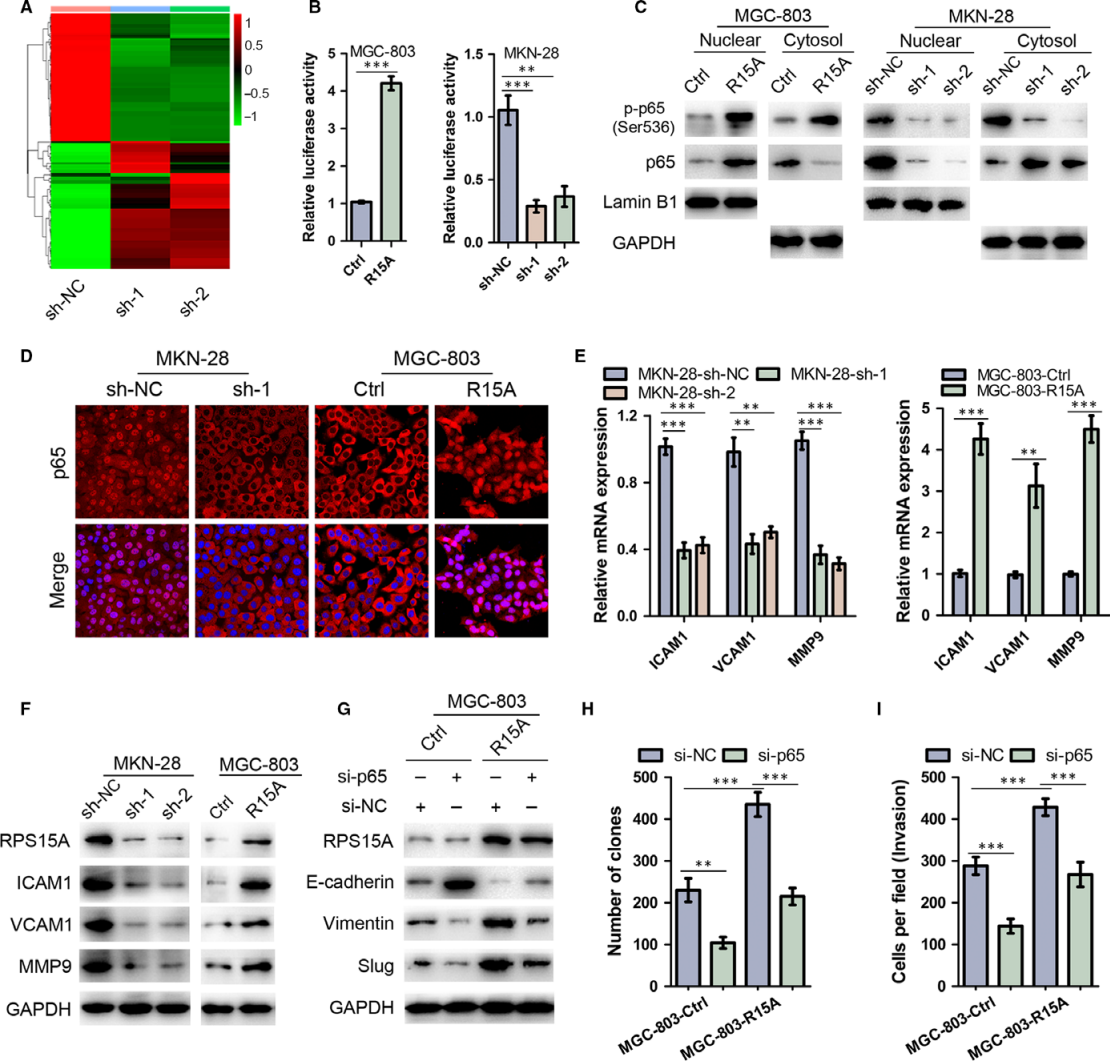
RPS15A promotes EMT through NF‐κB signalling. A, Gene expression profiles from RPS15A‐depleted MKN‐28 and control cells. The genes are shaded with green, black or red in the heat map to indicate low, intermediate or high expression respectively. B, MGC‐803 and MKN‐28 cells with modified RPS15A expression were transfected with NF‐κB luciferase reporter plasmid, and subjected to luciferase reporter assay. Data were normalized against Renilla luciferase activity and represented as mean ± SD. **P < 0.01; ***P < 0.001 by Student's *t* test. C, Western blotting analysis of nuclear and cytoplasmic p‐p65 and p65 protein expression in the indicated cells with modified RPS15A expression. Lamin B1 or GAPDH was used as internal control respectively. D, Subcellular p65 localization in indicated cells was assessed by immunofluorescence assay. p65 was stained in red colour, and the nuclei were stained with DAPI in blue colour. (E‐F) qRT‐PCR and Western blotting analyses of the expression of the NF‐κB signalling target genes (ICAM1, VCAM1 and MMP9) in indicated GC cells with modified RPS15A expression. GAPDH was used as an internal control. **P < 0.01; ***P < 0.001 by Student's *t* test. G, RPS15A‐overexpressed and control MGC‐803 cells were transfected with si‐p65, and then subjected to Western blotting analysis of EMT markers (E‐cadherin, vimentin and slug). GAPDH was used as an internal control. (H‐I) RPS15A‐overexpressed and control MGC‐803 cells were transfected with si‐p65, and then subjected to colony formation (H) and transwell invasion assays (I). **P < 0.01; ***P < 0.001 by Student's *t* test

To further explore the molecular mechanism by which RPS15A promotes GC development, RNA‐seq analysis was introduced to obtain the transcriptional profiles of RPS15A‐depleted MKN‐28 and control cells. Subsequent KEGG enrichment analysis identified the NF‐κB as the top‐ranked signalling pathway (Figure 5A). We then performed luciferase reporter assay to validate above findings, and found that the NF‐κB transcriptional activity was significantly activated by RPS15A overexpression, whereas markedly inhibited by RPS15A depletion (Figure 5B). Activation of NF‐κB pathway is known to require translocation of the p65 subunit from the cytoplasm to the nucleus.^8^, ^19^ To validate the results of luciferase reporter assay, we examined the nuclear localization of p65 protein by Western blotting analysis of fractionated proteins or by immunofluorescence assay in GC cell lines with modified RPS15A expression. And the results suggested that the nuclear levels of activated form p‐p65 were significantly higher in RPS15A‐overexpressed MGC‐803 cells than control cells (Figure 5C). Similarly, RPS15A overexpression also resulted in substantial nuclear accumulation of p65 (Figure 5C,D). RPS15A knockdown in MKN‐28 cells, on the other hand, led to decreased expression of nuclear p‐p65 and p65 protein (Figure 5C,D). Furthermore, we observed that both the mRNA and protein levels of a collection of NF‐κB downstream effectors that are central co‐ordinators of cancer cell metastasis, such as ICAM1, VCAM1 and MMP9, were decreased by RPS15A knockdown, but increased by RPS15A overexpression (Figure 5E,F). Taken together, these findings suggested a key role of RPS15A in transactivation of the NF‐κB signalling pathway.

To determine whether activation of NF‐κB is required for RPS15A‐induced EMT and GC progression, p65 was suppressed in RPS15A‐overexpressed MGC‐803 cells by siRNA. As shown in Figure 5G, the knockdown of p65 blocked the RPS15A‐induced changes in expression of E‐cadherin, vimentin and slug. Furthermore, the colony formation and transwell invasion assays indicated that the enhanced proliferation and invasive ability of MGC‐803 cells that resulted from RPS15A overexpression was also partially reversed by p65 knockdown (Figure 5H,I). Collectively, these data suggested that RPS15A promotes EMT through NF‐κB signalling.

### 3.6 RPS15A activates NF‐κB through the Akt/IKK‐β pathway

**Figure 6:**
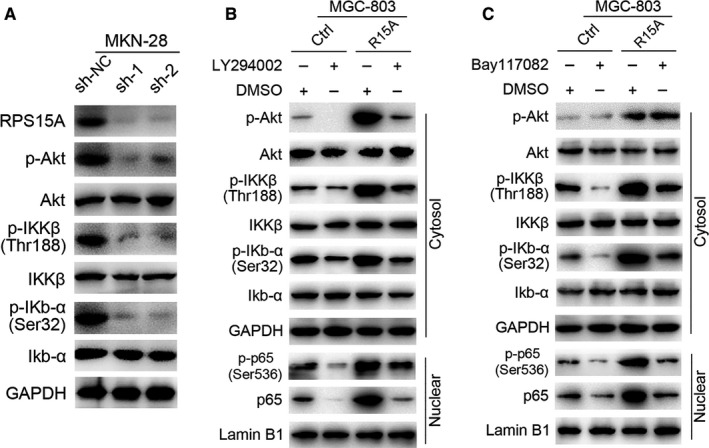
RPS15A activates NF‐κB through the Akt/IKK‐β pathway. A, Western blotting analysis of the expression of indicated proteins in RPS15A‐knockdown and control MKN‐28 cells. GAPDH was used as an internal control. B, RPS15A‐overexpressed or control MGC‐803 cells were treated with LY294002 (50 μmol/L) or DMSO for 2 h. Then indicated protein levels were assayed by Western blotting. Lamin B1 or GAPDH was used as an internal control respectively. C, RPS15A‐overexpressed or control MGC‐803 cells were treated with Bay117082 (20 μmol/L) or DMSO for 2 h. Then indicated protein levels were assayed by Western blotting. Lamin B1 or GAPDH was used as an internal control respectively.

NF‐κB activation is known to be mediated through the Akt/IKK‐β pathway.^20^, ^21^ Previous study has demonstrated that RPS15A is involved in the activation of the Akt pathway in glioblastoma.^15^ Thus, we hypothesized that RPS15A might activate NF‐κB through the Akt/IKK‐β pathway in GC cells. To test this, we first examined the effect of modified RPS15A on p‐Akt and total Akt protein. The results revealed that a significant decrease of p‐Akt, but not total Akt, was observed in RPS15A‐depleted MKN‐28 compared to control cells (Figure 6A). In contrast, RPS15A overexpression significantly increased p‐Akt expression levels (Figure 6A). A key kinase for NF‐κB activation is IKK‐β, which releases subunit p65 from IkB‐α and facilitates its nuclear translocation.^19^ We then investigated whether RPS15A affects the status of IKK‐β and IkB‐α in GC cells. The results suggested that RPS15A depletion decreased the abundance of both p‐IKK‐β (at Thr188) and p‐IkB‐α (at Ser32), whereas total IKK‐β and IkB‐α levels were unchanged (Figure 6A). In contrast, up‐regulation of RPS15A led to increased phosphorylation of IKK‐β and IkB‐α (Figure 6B). Collectively, RPS15A activates the Akt/IKK‐β pathway in GC cells.

To further validate the involvement of Akt and IKK‐β in RPS15A‐induced NF‐κB activation, we adopted a pharmacologic approach. We treated the RPS15A‐overexpressed MGC‐803 and control cells with Akt inhibitor LY294002 (50 μmol/L) or IKK inhibitor Bay117082 (20 μmol/L) for 2 hours. As expected, both LY294002 and Bay117082 neutralized the activation of Akt/IKK‐β signalling induced by RPS15A overexpression (Figure 6C), further confirming the crucial role of RPS15A in the activation of Akt/IKK‐β pathway. Importantly, we identified that both LY294002 and Bay117082 treatment significantly blocked the p65 and p‐p65 nuclear translocation that resulted from RPS15A overexpression (Figure 6C), suggesting that the RPS15A‐induced NF‐κB activation is indeed mediated by the Akt/IKK‐β pathway.

## 4 DISCUSSION

**Figure 7:**
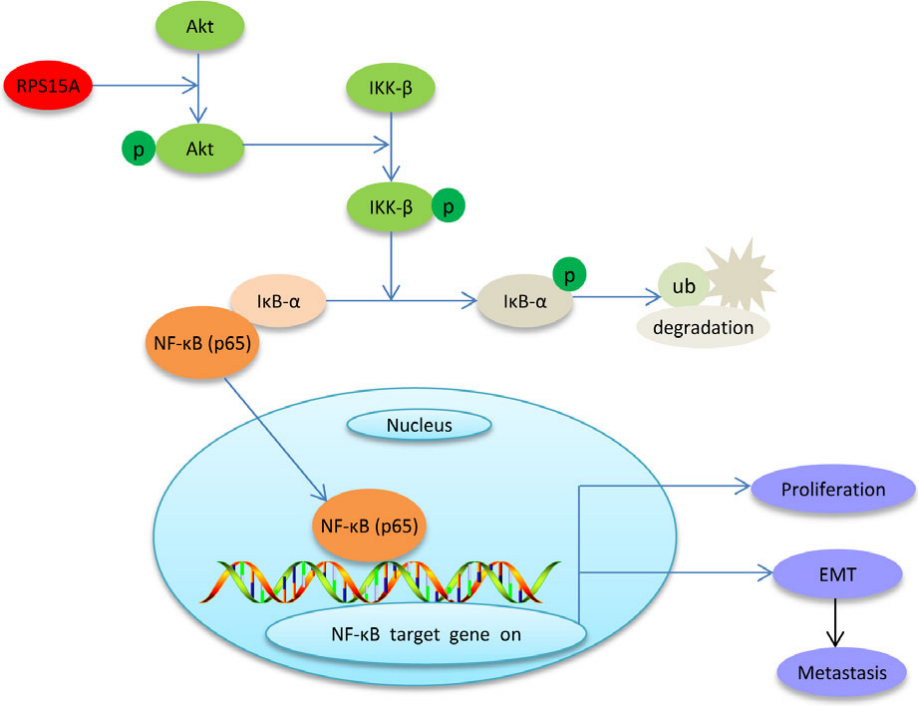
Proposed signalling pathways involved in RPS15A‐induced GC metastasis. RPS15A activates the Akt/IKK‐β signalling axis, and then triggers IκB‐α phosphorylation and degradation. Subsequently, the NF‐κB (p65) is released from the p65/IκB‐α complex and translocases to the nucleus, and consequently promotes EMT and GC metastasis.

The promotive role of RPS15A in tumour progression has been increasingly recognized recent years, whereas the underlying molecular mechanism remains largely unclear. In this study, we highlighted the clinical significance and oncogenic role of RPS15A in GC evolution. Furthermore, we provided evidence that RPS15A activates the NF‐κB pathway through Akt/IKK‐β signalling axis, and consequently promotes EMT and GC metastasis (Figure 7).

Distant metastasis and invasion of cancer are responsible for more than 90% of cancer‐related deaths.^22^ Metastasis is a complex process that involves multiple sequential steps, including invasion of cancer cells into surrounding tissue, intravasation, survival in the circulation, arrest at distant organ sites, extravasation and growth of a macroscopic secondary tumour in distant organs.^23^ Although numerous genes involved in GC metastasis were identified, metastatic mechanism of GC remains poorly understood. Previous studies have revealed that RPS15A is aberrantly overexpressed in various types of cancer,^14^, ^17^, ^24^ and its expression could promote cell proliferation, tumour angiogenesis and inhibit cell cycle arrest and apoptosis.^15^, ^16^, ^24^, ^25^, ^26^, ^27^, ^28^ However, the potential effect of RPS15A on cell invasion and metastasis has not been investigated. In parallel with these researches, we confirmed the up‐regulation of RPS15A in 40 GC samples compared to paired normal tissues. We also evaluated the expression pattern of RPS15A with a tissue microarray consisting of 186 GC samples, and found that elevated expression of RPS15A is correlated to aggressive characteristics and poor prognosis. In addition, by gain‐ and loss‐of‐function studies, we demonstrated that RPS15A promotes the proliferation, migration and invasion of GC cells both in vitro and in vivo. Thus our data would be helpful to augment the tumour biology of RPS15A.

The initial stage of metastatic progression is essentially dependent on EMT.^3^, ^5^ Nevertheless, a more in‐depth understanding of the factors promoting EMT in GC and GC metastasis is urgently needed in order to identify specific biomarkers for improving GC prognosis and treatment. Here, by using qRT‐PCR, Western blotting and immunofluorescence assays, we report that cells with high levels of RPS15A expressed low levels of E‐cadherin, and high levels of vimentin and slug, suggesting that RPS15A may be a potent inducer of EMT, which may result in more invasive and metastatic GC cells.

Tumour invasion and metastasis are complex, multistep processes underlain by genetic and/or epigenetic changes within probably a fraction of malignant cells in the tumour.^29^, ^30^ Molecular mechanisms underlying the EMT programme and distant metastasis of GC have been intensively studied. Cellular signalling pathways, including the Wnt/β‐catenin, TGF‐β, Notch, Hedgehog and NF‐κB, have been demonstrated to be aberrantly activated and play vital roles in the development and progression of EMT and GC progression.^31^, ^32^ By RNA‐seq and luciferase reporter assays, we identified the NF‐κB pathway may be the potential effector of RPS15A. This hypothesis was subsequently confirmed by the nuclear translocation and phosphorylation of p65, increased transactivation of NF‐κB reporter and up‐regulation of target genes of this pathway at both mRNA and protein levels. Furthermore, we demonstrated NF‐κB signalling pathway as a mediator involved in RPS15A‐induced EMT and GC metastasis, as p65 knockdown partially blocked the expression changes of EMT markers and altered cell proliferation and motility that resulted from RPS15A overexpression. Thus, we could reasonably assume that RPS15A facilitates GC metastasis via NF‐κB‐regulated EMT.

The systematic control of the NF‐κB pathway relies on the unique property of its major inhibitor, IκB‐α.^33^ Upon activation of the pathway, the IKK kinase complex phosphorylates IκB‐α, inducing its degradation and promoting the release and relocalization of NF‐κB to the nucleus, where it exerts its role in gene expression.^34^, ^35^ Akt/IKK‐β axis, known as the upstream signalling of NF‐κB pathway, plays vital roles in promoting tumour cell survival, invasive behaviour and chemosensitivity in various malignancies.^9^, ^21^, ^36^ A previous study has shown that RPS15A depletion leads to decrease in p‐Akt level in glioblastoma cells.^15^ In this study, to investigate the mechanism by which RPS15A induces p65 nuclear translocation, we adopted Akt inhibitor LY294002 or IKK inhibitor Bay117082. Our data suggested that both LY294002 and Bay117082 significantly interfered with the activation of Akt/IKK‐β signalling induced by RPS15A overexpression. Thus we speculated that RPS15A activates NF‐κB through the Akt/IKK‐β pathway.

In conclusion, our study demonstrated that elevated expression of RPS15A is closely correlated with poor prognosis of GC patients and promotes EMT and GC progression via Akt/IKK‐β/NF‐κB signalling pathway, thus possibly providing a promising candidate for treatment against GC metastasis.

## CONFLICT OF INTEREST

The authors declare that they have no conflict of interest.

## References

[1] Van Cutsem E , Sagaert X , Topal B , et al. Gastric cancer. Lancet. 2016;388:2654‐2664. 27156933 10.1016/S0140-6736(16)30354-3

[2] Karimi P , Islami F , Anandasabapathy S , et al. Gastric cancer: descriptive epidemiology, risk factors, screening, and prevention. Cancer Epidemiol Biomark Prev. 2014;23:700‐713. 10.1158/1055-9965.EPI-13-1057 PMC4019373 24618998

[3] Sleeman JP , Thiery JP . SnapShot: the epithelial‐mesenchymal transition. Cell. 2011;145:162.e1. 21458675 10.1016/j.cell.2011.03.029

[4] Liu CC , Cai DL , Sun F , et al. FERMT1 mediates epithelial‐mesenchymal transition to promote colon cancer metastasis via modulation of beta‐catenin transcriptional activity. Oncogene. 2017;36:1779‐1792. 27641329 10.1038/onc.2016.339

[5] Gonzalez DM , Medici D . Signaling mechanisms of the epithelial‐mesenchymal transition. Sci Signal. 2014;7:re8. 25249658 10.1126/scisignal.2005189 PMC4372086

[6] Chen D , Cao G , Qiao C , et al. Alpha B‐crystallin promotes the invasion and metastasis of gastric cancer via NF‐kappaB‐induced epithelial‐mesenchymal transition. J Cell Mol Med. 2018;22:3215‐3222. 29566309 10.1111/jcmm.13602 PMC5980171

[7] Huber MA , Azoitei N , Baumann B , et al. NF‐kappaB is essential for epithelial‐mesenchymal transition and metastasis in a model of breast cancer progression. J Clin Invest. 2004;114:569‐581. 15314694 10.1172/JCI21358 PMC503772

[8] Scheidereit C . IkappaB kinase complexes: gateways to NF‐kappaB activation and transcription. Oncogene. 2006;25:6685‐6705. 17072322 10.1038/sj.onc.1209934

[9] Hayden MS , Ghosh S . NF‐kappaB, the first quarter‐century: remarkable progress and outstanding questions. Genes Dev. 2012;26:203‐234. 22302935 10.1101/gad.183434.111 PMC3278889

[10] Jimenez L , Becerra A , Landa A . Cloning, expression and partial characterization of a gene encoding the S15a ribosomal protein of *Taenia solium* . Parasitol Res. 2004;92:414‐420. 14760523 10.1007/s00436-003-1021-4

[11] Shen X , Valencia CA , Szostak JW , et al. Scanning the human proteome for calmodulin‐binding proteins. Proc Natl Acad Sci USA. 2005;102:5969‐5974. 15840729 10.1073/pnas.0407928102 PMC1087907

[12] Bonham‐Smith PC , Oancia TL , Moloney MM . Cytoplasmic ribosomal protein S15a from *Brassica napus*: molecular cloning and developmental expression in mitotically active tissues. Plant Mol Biol. 1992;18:909‐919. 1581568 10.1007/BF00019205

[13] Akiyama N , Matsuo Y , Sai H , et al. Identification of a series of transforming growth factor beta‐responsive genes by retrovirus‐mediated gene trap screening. Mol Cell Biol. 2000;20:3266‐3273. 10757810 10.1128/mcb.20.9.3266-3273.2000 PMC85620

[14] Zhao X , Shen L , Feng Y , et al. Decreased expression of RPS15A suppresses proliferation of lung cancer cells. Tumor Biol. 2015;36:6733‐6740. 10.1007/s13277-015-3371-9 25833696

[15] Yao Y , Liu Y , Lv X , et al. Down‐regulation of ribosomal protein S15A inhibits proliferation of human glioblastoma cells in vivo and in vitro via AKT pathway. Tumor Biol. 2016;37:4979‐4990. 10.1007/s13277-015-4323-0 26537582

[16] Zhang C , Fu J , Xue F , et al. Knockdown of ribosomal protein S15A induces human glioblastoma cell apoptosis. World J Surg Oncol. 2016;14:129. 27130037 10.1186/s12957-016-0891-8 PMC4850639

[17] Chen J , Wei Y , Feng Q , et al. Ribosomal protein S15A promotes malignant transformation and predicts poor outcome in colorectal cancer through misregulation of p53 signaling pathway. Int J Oncol. 2016;48:1628‐1638. 26847263 10.3892/ijo.2016.3366

[18] Liu C , Yue B , Yuan C , et al. Elevated expression of Thoc1 is associated with aggressive phenotype and poor prognosis in colorectal cancer. Biochem Biophys Res Commun. 2015;468:53‐58. 26545775 10.1016/j.bbrc.2015.10.166

[19] Hinz M , Scheidereit C . The IkappaB kinase complex in NF‐kappaB regulation and beyond. EMBO Rep. 2014;15:46‐61. 24375677 10.1002/embr.201337983 PMC4303448

[20] Kuo MT , Liu Z , Wei Y , et al. Induction of human MDR1 gene expression by 2‐acetylaminofluorene is mediated by effectors of the phosphoinositide 3‐kinase pathway that activate NF‐kappaB signaling. Oncogene. 2002;21:1945‐1954. 11960367 10.1038/sj.onc.1205117

[21] Gao S , Sun Y , Zhang X , et al. IGFBP2 activates the NF‐kappaB pathway to drive epithelial‐mesenchymal transition and invasive character in pancreatic ductal adenocarcinoma. Cancer Res. 2016;76:6543‐6554. 27659045 10.1158/0008-5472.CAN-16-0438 PMC5315491

[22] Chaffer CL , Weinberg RA . A perspective on cancer cell metastasis. Science. 2011;331:1559‐1564. 21436443 10.1126/science.1203543

[23] Steeg PS . Tumor metastasis: mechanistic insights and clinical challenges. Nat Med. 2006;12:895‐904. 16892035 10.1038/nm1469

[24] Zhang Y , Zhang G , Li X , Li B , Zhang X . The effect of ribosomal protein S15a in lung adenocarcinoma. PeerJ. 2016;4:e1792. 26989627 10.7717/peerj.1792 PMC4793315

[25] Guo P , Wang Y , Dai C , et al. Ribosomal protein S15a promotes tumor angiogenesis via enhancing Wnt/beta‐catenin‐induced FGF18 expression in hepatocellular carcinoma. Oncogene. 2018;37:1220‐1236. 29242604 10.1038/s41388-017-0017-y

[26] Li G , Zhang L , Liu J , et al. shRNA‐mediated RPS15A silencing inhibits U937 acute myeloid leukemia cell proliferation and enhances apoptosis. Mol Med Rep. 2016;13:4400‐4406. 27035327 10.3892/mmr.2016.5064

[27] Zhang C , Zhang T , Song E , et al. Ribosomal protein S15A augments human osteosarcoma cell proliferation in vitro. Cancer Biother Radiopharm. 2014;29:451‐456. 25409460 10.1089/cbr.2014.1698 PMC4267417

[28] Xu M , Wang Y , Chen L , et al. Down‐regulation of ribosomal protein S15A mRNA with a short hairpin RNA inhibits human hepatic cancer cell growth in vitro. Gene. 2014;536:84‐89. 24334120 10.1016/j.gene.2013.11.075

[29] Eccles SA , Welch DR . Metastasis: recent discoveries and novel treatment strategies. Lancet. 2007;369:1742‐1757. 17512859 10.1016/S0140-6736(07)60781-8 PMC2214903

[30] Gupta GP , Massague J . Cancer metastasis: building a framework. Cell. 2006;127:679‐695. 17110329 10.1016/j.cell.2006.11.001

[31] Peng Z , Wang CX , Fang EH , et al. Role of epithelial‐mesenchymal transition in gastric cancer initiation and progression. World J Gastroenterol. 2014;20:5403‐5410. 24833870 10.3748/wjg.v20.i18.5403 PMC4017055

[32] Huang L , Wu RL , Xu AM . Epithelial‐mesenchymal transition in gastric cancer. Am J Transl Res. 2015;7:2141‐2158. 26807164 PMC4697696

[33] Ferreiro DU , Komives EA . Molecular mechanisms of system control of NF‐kappaB signaling by IkappaBalpha. Biochemistry. 2010;49:1560‐1567. 20055496 10.1021/bi901948j PMC2865148

[34] Smale ST . Hierarchies of NF‐kappaB target‐gene regulation. Nat Immunol. 2011;12:689‐694. 21772277 10.1038/ni.2070 PMC3169328

[35] Miyamoto S . Nuclear initiated NF‐kappaB signaling: NEMO and ATM take center stage. Cell Res. 2011;21:116‐130. 21187855 10.1038/cr.2010.179 PMC3193401

[36] Azijli K , Weyhenmeyer B , Peters GJ , et al. Non‐canonical kinase signaling by the death ligand TRAIL in cancer cells: discord in the death receptor family. Cell Death Differ. 2013;20:858‐868. 23579241 10.1038/cdd.2013.28 PMC3679459

