# Supplementary material for: RPS15A promotes gastric cancer progression via activation of the Akt/IKK‐β/NF‐κB signalling pathway
Source: J Cell Mol Med. 2019 Jan 19;23(3):2207–18. doi: 10.1111/jcmm.14141 (PMC6378197; doi:10.1111/jcmm.14141)
Supplement: Supplementary file 1 [file JCMM-23-2207-s001.docx]

**Table S1 Sequence of primers for Quantitative reverse transcription-PCR**

| Gene | Forward primer (5’------3’) | Reverse primer(5’------3’) |
| --- | --- | --- |
| RPS15A | AATGTCCTGGCAGATGCTCTCAAG | GAGCACGGCCTAATAAGCACCTG |
| E-cadherin | AGGCCAAGCAGCAGTACATT | CATTCACATCCAGCACATCC |
| Vimentin | CGAAACTTCTCAGCATCACG | GCAGAAAGGCACTTGAAAGC |
| Slug | TCTTCACTCCGAAGCCAAAT | TCTGTGGGTGTGTGTGTGTG |
| ICAM1 | CCGGAAGGTGTATGAACTG | CAGTTCATACACCTTCCGG |
| VCAM1 | TTCTGTGCCCACAGTAAGG | GCAGCTTTGTGGATGGATTC |
| MMP9 | CCTCTGGAGGTTCGACGTGA | TAGGCTTTCTCTCGGTACTGGAA |
| GAPDH | AGAAGGCTGGGGCTCATTTG | AGGGGCCATCCACAGTCTTC |
